# 17-4 PH Steel Parts Obtained through MEX and PBF-LB/M Technologies: Comparison of the Structural Properties

**DOI:** 10.3390/ma17122801

**Published:** 2024-06-07

**Authors:** Katarzyna Jasik, Lucjan Śnieżek, Janusz Kluczyński, Jakub Łuszczek, Krzysztof Grzelak, Bartłomiej Sarzyński, Ireneusz Szachogłuchowicz

**Affiliations:** Institute of Robots & Machine Design, Faculty of Mechanical Engineering, Military University of Technology, Gen. S. Kaliskiego 2, 00-908 Warsaw, Poland; katarzyna.jasik@wat.edu.pl (K.J.); lucjan.sniezek@wat.edu.pl (L.Ś.); jakub.luszczek@wat.edu.pl (J.Ł.); krzysztof.grzelak@wat.edu.pl (K.G.); bartlomiej.sarzynski@wat.edu.pl (B.S.); ireneusz.szachogluchowicz@wat.edu.pl (I.S.)

**Keywords:** additive manufacturing, metal extrusion, laser beam powder bed fusion of metals, steel 17-4 PH

## Abstract

The material extrusion (MEX) method utilizing highly filled metal filament presents an alternative to advanced additive metal manufacturing technologies. This process enables the production of metal objects through deposition and sintering, which is particularly attractive compared to powder bed fusion (PBF) technologies employing lasers or high-power electron beams. PBF requires costly maintenance, skilled operators, and controlled process conditions, whereas MEX does not impose such requirements. This study compares research on 17-4 PH steel manufactured using two different commercially available techniques: MEX and powder bed fusion with laser beam melting (PBF-LB/M). This research included assessing the density of printed samples, analyzing surface roughness in two printing planes, examining microstructure including porosity and density determination, and measuring hardness. The conducted research aimed to determine the durability and quality of the obtained samples and to evaluate their strength. The research results indicated that samples produced using the PBF-LB/M technology exhibited better density and a more homogeneous structure. However, MEX samples exhibited better strength properties (hardness).

## 1. Introduction

Contemporary additive manufacturing (AM) technology plays a significant role in various industrial fields. It offers the capability to create three-dimensional objects by depositing material layers in a computationally controlled manner. Unlike traditional manufacturing methods such as machining or casting, 3D printing brings several advantages. It enables rapid prototyping and the fabrication of complex geometric elements, design flexibility, material waste reduction, and the elimination of the need for molds and dyes characteristic of conventional production methods [1,2,3].

Three-dimensional printing technology enables object creation by precisely layering material onto itself. There are numerous 3D printing techniques, each employing different layering methods and materials. Some of the most popular techniques include material extrusion (MEX), powder bed fusion (PBF), direct energy deposition (DED), and material jetting (MJ) [4].

Initially, various polymers, mainly polylactic acid (PLA) [5,6,7,8], polyamide-based (PA) [9,10,11], and acrylonitrile–butadiene–styrene copolymer (ABS) [12], were the materials of choice for AM. These materials were favored for their ease of printing, availability, and relatively low costs. PLA, being a biodegradable polymer, gained popularity due to its environmental friendliness. As technology advanced and more sophisticated applications were pursued, other material groups, such as ceramics [13,14,15] and metals [16,17], began to be explored. In the early stages of metal printing development, a group of metal printing techniques called PBF-LB/M (laser-based metal powder bed fusion) was utilized, with SLM (selective laser melting) being one of the leading solutions [18]. This process involves melting layers of metal powder using a concentrated laser beam, precisely melting the material in a small area, and creating successive layers of the object. The quality and properties of parts produced in this manner are comparable to parts manufactured using traditional methods [19]. However, this technique has certain drawbacks. One of the main disadvantages is its high cost [20]. Both investments in advanced printing equipment and the price of printing materials can be significantly higher than with other methods. Additionally, the PBF-LB/M process requires meticulous control of printing parameters, such as temperature and laser scanning speed, which may require specialized training for machine operators [21]. Therefore, in recent years, materials have been developed that enable metal printing using MEX techniques, including Fused Deposition Modeling (FDM) and Fused Filament Fabrication (FFF). MEX techniques are currently the most popular 3D printing techniques due to their advantages, including low cost, a high availability of equipment and materials, and relatively easy operation without the need for high-power lasers or loose powders [22]. Implementing metallic materials in the MEX technique requires the use of a special composite filament containing metallic powder embedded in a polymer matrix. However, to obtain parts of pure metal, the 3D-printed elements must undergo a process called removal of the catalytic binder (debinding), followed by sintering to remove the binding phase and densify the parts. Due to the relatively recent emergence of metallic materials intended for MEX printing, there is limited research available [23]. The first and currently most popular material is 316L steel. The authors of [24] focused on analyzing the influence of printing direction on the performance properties of elements made from BASF 316L material. Static tensile tests revealed that the samples exhibited a similar failure process, except for tensile strength and elongation at break. Meanwhile, Quarto et al. [25] researched selected printing parameters to improve the performance properties of printed parts, minimize their porosity, and investigate dimensional shrinkage. Kedziora et al. [26] examined the strength properties of 316L steel printed using MEX technology and compared the results with those obtained for 316L steel printed using PBF-LB/M technology. A significant decrease in tensile strength and fatigue strength of samples printed using MEX was observed compared to those printed using PBF-LB/M technology. Also, the authors of [27] noted that MEX-printed samples exhibited lower yield strength, tensile strength, and Young’s modulus. However, in the past three years, a new material, 17-4 PH steel, has emerged on the market, which features better strength properties than 316L steel. Due to the recent emergence of 17-4 PH steel for MEX printing, there is limited research available. The available research focuses on assessing the impact of printing parameters on individual properties, but there is a lack of studies comparing 17-4 PH steel printed using different methods. Fazzini et al. [28] focused on optimizing the MEX printing process using filaments to maximize the mechanical properties of sintered metal products by controlling printing parameters, such as raster angle, which affect Young’s modulus and elongation at break. Meanwhile, Atatreh et al. [29] focused on the influence of infill patterns on the mechanical properties of metal parts produced by MEX using 17-4 PH stainless steel.

Considering the benefits of 3D metal printing using MEX technology, including the simplicity of the printing process and the low costs of materials and equipment, further research is needed on the processes and possibilities of improving the material’s functional properties, particularly in comparison to the currently most popular metal 3D printing techniques, such as PBF-LB/M. Therefore, this article aims to assess the potential of MEX techniques as an alternative to more expensive AM methods like PBF-LB/M, as well as analyzing the main advantages and disadvantages of both technologies. The article compares the microstructure, hardness, and surface roughness of two metal parts printed using MEX and PBF-LB/M techniques.

## 2. Materials and Methods

### 2.1. MEX Manufacturing

To produce samples using the MEX technique, Ultrafuse 17-4 PH filament from BASF (BASF, Ludwigshafen am Rhein, Germany) was utilized. This material takes the form of a composite, comprising a polymeric binder and densely packed metallic powder. Further thermochemical processing is required for this type of material to obtain fully metallic parts. It is printable using a standard material extrusion-based printer. Initially, cubic samples with a length of 15 mm were printed on a Prusa i3 MK3 3D printer (Prusa Research, Prague, Czech Republic). The printing parameters are presented in Table 1.

**Figure 1:**
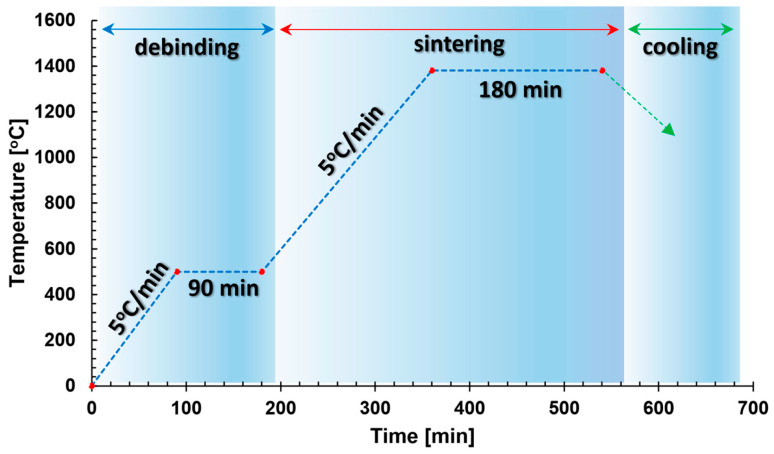
Thermal cycle of debinding and sintering process of 17-4 PH BASF Ultrafuse material [30].

The printed parts, referred to as “green parts” underwent a catalytic debinding process aimed at the preliminary removal of the polymeric binder. As a result, a “brown part” was obtained, which was then subjected to a sintering process, ultimately eliminating the secondary binder and resulting in fully densified metallic parts (Figure 1). This processing was carried out directly by the material manufacturer as an external service.

### 2.2. PBF-LB/M Manufacturing

To produce samples using the PBF-LB/M technique, stainless steel 17-4 PH powder from SLM Solutions (SLM Solutions Group AG, Estlandring 4, 23560 Lübeck, Germany) was utilized. The powder consisted of spherical particles with diameters ranging from 15 to 75 μm. The chemical composition of the powder is detailed in Table 2.

The samples had a cubic shape with a length of 15 mm along with a supporting layer with a height of 3 mm. The samples were printed on an SLM 125HL device (SLM Solutions AG, Lübeck, Germany) with the use of process parameters (shown in Table 3), which influence the volumetric energy density [31]

The energy density was calculated based on the following formula:

$$V_{ED}=\frac{P_{L}}{V_{s}*h_{d}*L_{T}}$$

V_ED_—volumetric energy density (J/mm^3^);

P_l_—laser power (W);

v_s_—exposure velocity (mm/s);

h_d_—hatching distance (mm);

L_T_—layer thickness (mm).

During the printing process, adjustments were made to the platform height of the device, and the first layer of powder, known as the “zero level”, was applied. A laser with a wavelength of 1080 nm was utilized throughout the process.

### 2.3. Preparation of the Printed Samples Involved

**Figure 2:**
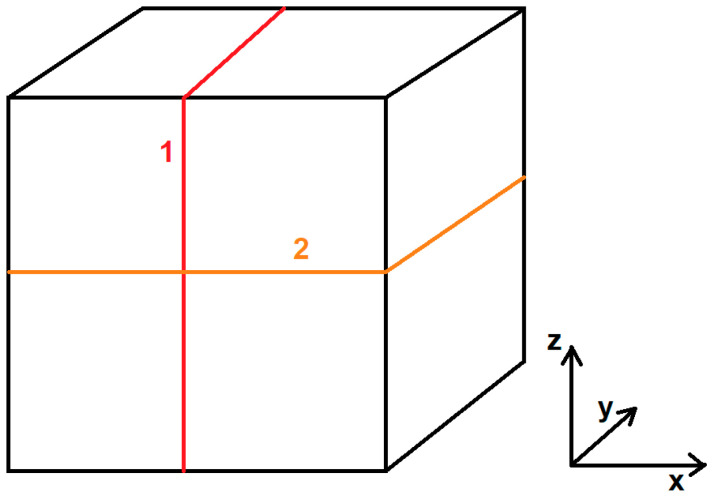
Sample cutting planes: 1—YZ plane, along the printed layers; 2—XY plane, along the direction of layer deposition.

**Figure 3:**
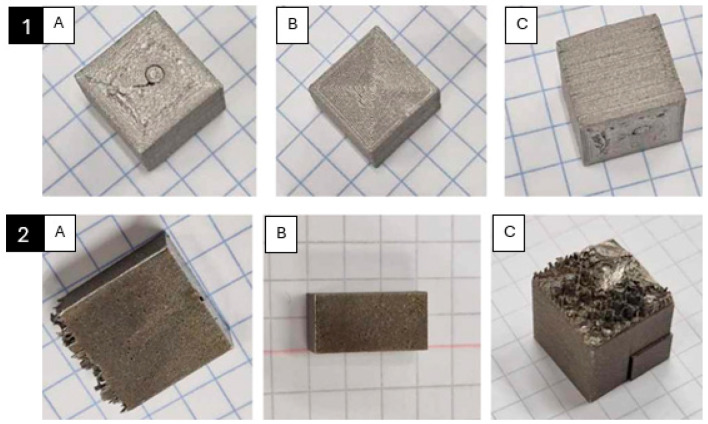
Sample PBF-LB/M 1—after processing: (**A**) cut along the printed layers (YZ plane); (**B**) cut along the direction of layer deposition (XY plane); (**C**) entire sample; MEX 2—after processing: (**A**) cut along the printed layers (YZ plane); (**B**) cut along the direction of layer deposition (XY plane); (**C**) entire sample.

In the next step, samples were prepared for microstructural analysis. For this purpose, a portion of the samples was cut parallel to the printed layers. The cutting planes are shown in Figure 2. The cut samples are presented in Figure 3.

**Figure 4:**
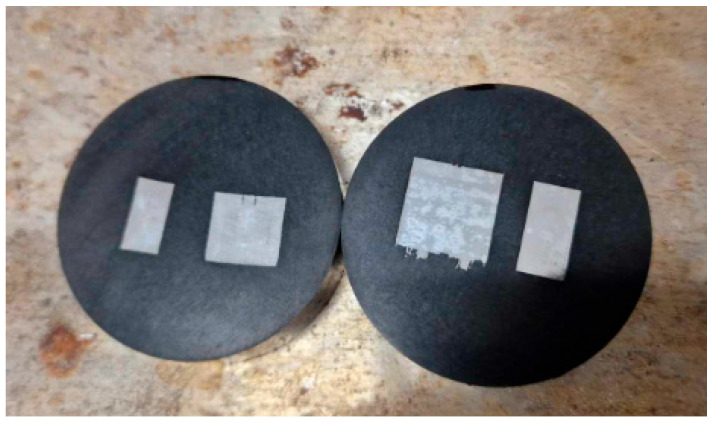
Samples prepared for microstructural analysis.

Next, the samples were embedded and ground with sandpaper of the following grits: 320, 500, 800, 1000, and 1200, and polished using a neoprene cloth (Op-Chem) with the addition of water and OP-S reagent. In the final stage, the samples underwent etching, which was conducted in a digestion unit using an acetic glycerol solution as the etchant. The exact composition of the solution is presented in Table 4. Figure 4 shows the samples after etching.

### 2.4. Microstructural Investigation

Immediately after the completion of the etching process, samples were subjected to microstructural analysis to assess the quality of the microstructure and to check for the presence of any potential material imperfections such as porosity, voids, or cracks. For this purpose, the digital microscope Keyence VHX7000 (Keyence, Osaka, Japan) was used. This study considered two planes of PBF-LB/M and MEX samples—along the printed layers (YZ plane) and along the direction of layer deposition (XY plane).

Next, using the confocal microscope Olympus 4100 LEXT OLS 4100 (Olympus, Shinjuku, Tokyo, Japan) and dedicated software (Mountains Map, version 7.0), the porosity of the samples was examined. It was calculated based on the average number of grains in each sample. For each specimen, the porosity was analyzed in the central part of the metallographic section. All visible pores were marked in both analyzed planes. Porosity quantitative analysis was based on the captured microscopic images. It was carried out using Mountains Map 6 Software.

In the next step, additional density measurements were carried out using the laboratory scale AXIS ATA220 (Axis, Gdansk, Poland) to verify the results according to Archimedes’ principle.

Prior to the experiments, the surface was cleaned to improve wettability and minimize surface tension. As part of the method, the weight of the object placed on a tray under atmospheric conditions was first measured, followed by its immersion in distilled water at room temperature, after which the weight was measured again. Based on these measurements, the density of the object and the density of the liquid were calculated. This process was repeated three times for each sample. Subsequently, the average density was calculated, which was used to determine the porosity of the solid body according to the formula:

$$p=\frac{\rho_{c}-\rho_{t}}{\rho_{c}}*100\%$$

*p*—porosity [%];

ρ*_c_*—density of conventional material [g/cm^3^];

ρ*_t_*—density of test material [g/cm^3^].

This research was conducted for three different types of samples made of 17-4 PH steel: using a conventional method and 3D printing methods—PBF-LB/M and MEX techniques. The conventional sample was used as a reference sample, to which the porosity of the other two 3D-printed samples was compared. Subsequently, microhardness measurements were performed using the Vickers method on the HV0.1 scale according to the PN-EN ISO 6507-1 standard [32], using the Struers DuraScan 70 hardness tester (Struers, Ballerup, Denmark). A diamond indenter with a regular tetrahedral shape and a vertex angle of 120° was used for the measurements. Five measurements were taken for each sample, and two extreme results were excluded from further calculations. To avoid mutual influence, the distance between individual measurements was three times greater than the diameter of the indentation.

In the final stage, surface roughness measurements were performed using the Keyence VHX7000 digital microscope. The roughness was measured on unprocessed samples along their layers in the central part. Measurements were taken for two planes—the upper and the lateral.

## 3. Results and Discussion

All results presented are the average measurements of three samples for each type of printing.

### 3.1. Microstructural Investigation

**Figure 5:**
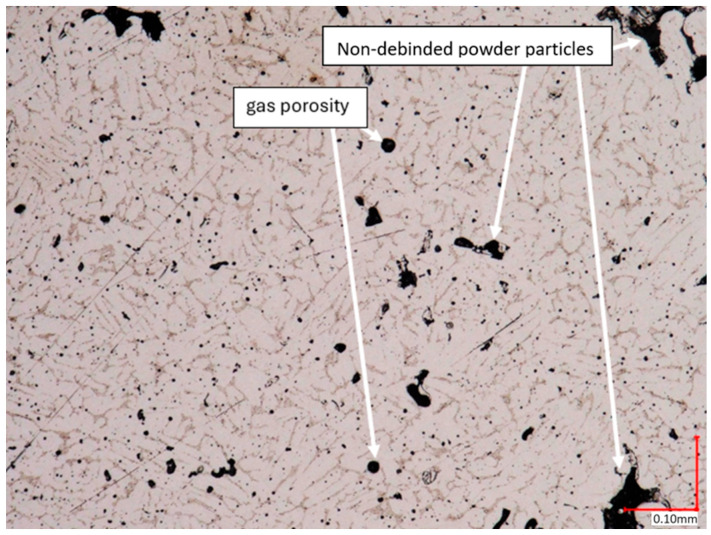
The microstructure of the MEX samples (cross-section taken in the XY plane).

**Figure 6:**
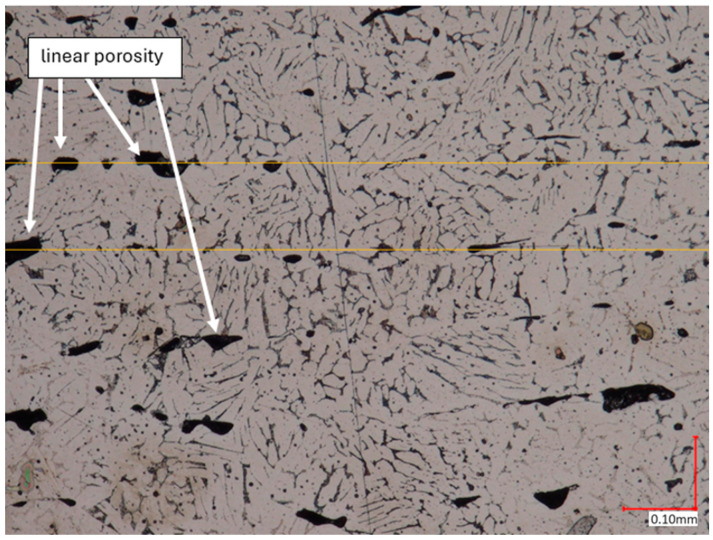
The microstructure of the MEX samples (cross-section taken in the YZ plane). One layer thickness marked by yellow lines.

**Figure 7:**
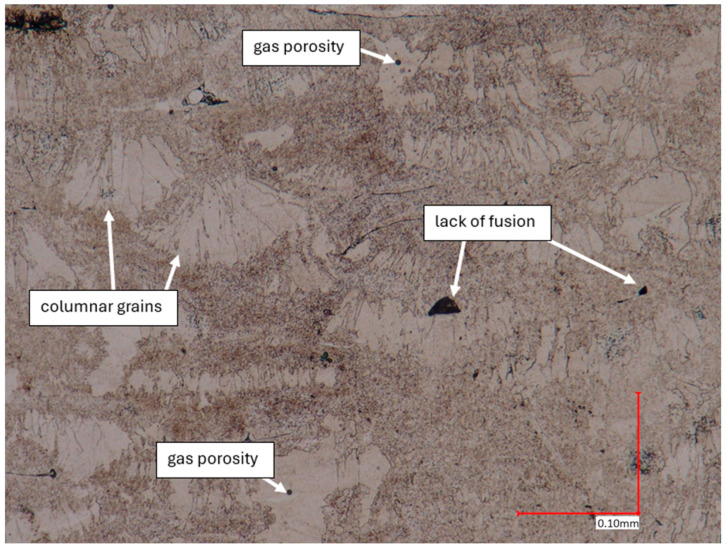
The microstructure of the PBF-LB/M samples (cross-section taken in the YZ plane).

**Figure 8:**
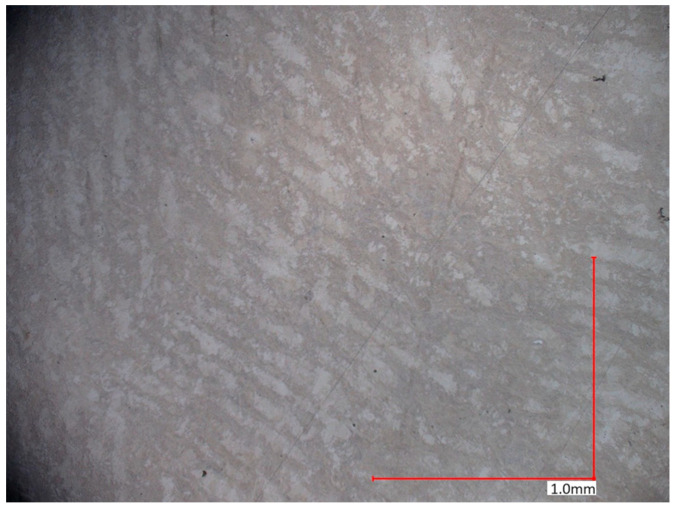
The microstructure of the PBF-LB/M samples (cross-section taken in the XY plane).

The microstructural analysis of samples produced by the MEX technique reveals the presence of void spaces with irregular shapes in both examined planes (Figure 5 and Figure 6). Notably, in Figure 5, the largest voids are observed between two adjacent deposited material paths, while the morphology exhibits a relatively regular pattern characterized by particles of uniform shapes and dimensions. Microscopic examination of samples fabricated via the PBF-LB/M method (Figure 7) unveils the existence of columnar grains, stemming from the specific crystallization kinetics during the solidification of the molten material layer in PBF-LB/M. The rapid cooling of the molten material layer induces swift solidification, resulting in the formation of these columnar grains. Additionally, the presence of voids within the structure is noted, with their irregular shapes possibly indicating localized areas of incomplete material fusion, a phenomenon often associated with the occurrence of porosity, known as lack of fusion (LOF). These findings suggest that the PBF-LB/M process engenders distinctive microstructural attributes, including orderly arranged alloy traces and potential porosity formation linked to lack of fusion. Another discernible form of porosity is linear porosity, observed at the periphery of the melt pool, attributed to the excessive spacing between successive paths traced by the laser scanner during the deposition of subsequent material layers. Moreover, circular voids akin to gas porosity are evident in microstructure images, signifying the presence of gas entrapment. This phenomenon arises due to chemical reactions occurring during the printing process or residual gas entrapment within the printed material, leading to the liberation of microscale gas pores. Such pores may arise from incomplete material fusion, inappropriate printing parameters, or chemical reactions among powder constituents. In the YZ plane (Figure 8), the structure demonstrates reduced porosity characterized by diminished void dimensions, alongside the discernible directionality of the exposure paths within the structure.

### 3.2. Porosity

**Figure 9:**
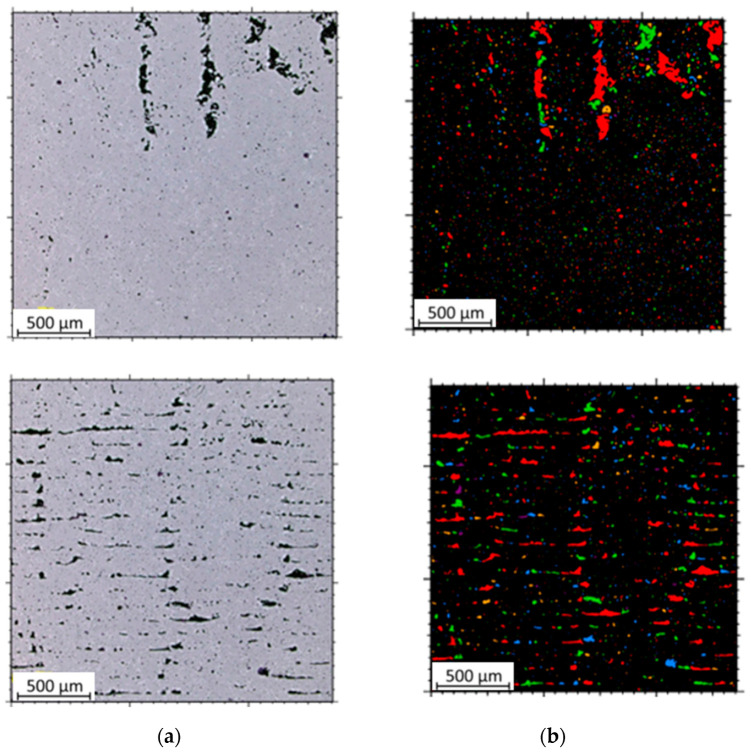
Tested microstructures and microstructures with marked porosity of samples printed using the MEX technique: (**a**) XY plane; (**b**) YZ plane.

**Figure 10:**
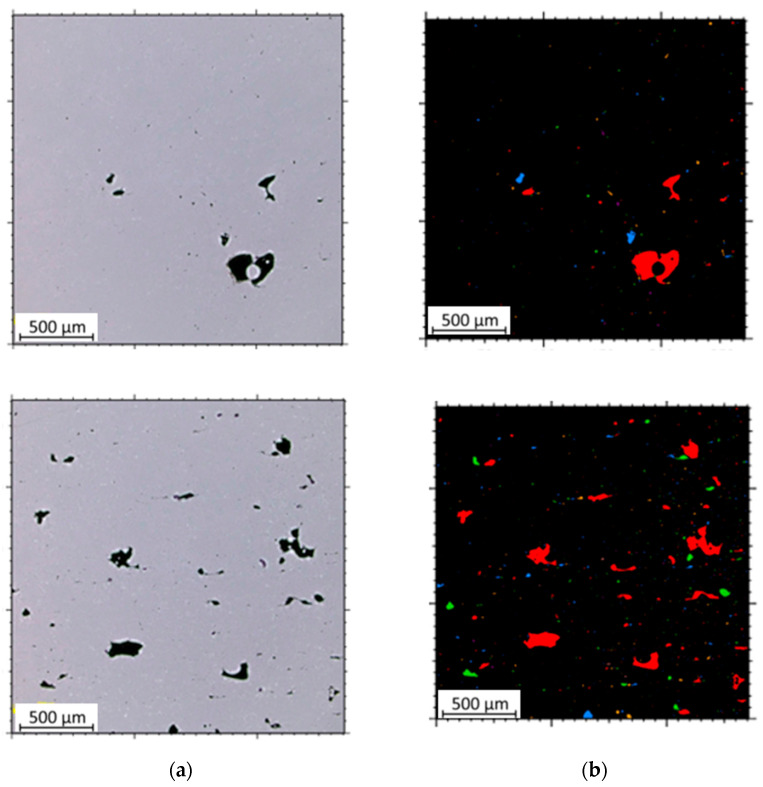
Tested microstructures and microstructures with marked porosity of samples printed using the PBF-LB/M technique: (**a**) XY plane; (**b**) YZ plane.

For each sample type—MEX and PBF-LB/M—three measurements of surface porosity were conducted for both XY and YZ planes. The result represents the average of these measurements. Below are single images from the study for each sample (Figure 9 and Figure 10).

Table 5 presents the results of the study. For PBF-LB/M samples, the porosity for the XY plane was 1.6%, and for the YZ plane, it was 2.7%. Samples printed using the MEX method exhibited higher porosity than those produced using the PBF-LB/M technique. For the XY plane, this value was almost three times higher than that obtained by the PBF-LB/M method, reaching 4.5%. The difference was not as significant for the YZ plane. The porosity of the MEX sample was 11% higher than that of the PBF-LB/M sample, amounting to 2.4%. Microhardness tests for MEX and PBF-LB/M samples were also conducted for two planes. The load during HV0.1 measurements was 4.9 N. The results are presented in Table 5.

### 3.3. Archimedes’ Method

The results of porosity and density for the three examined samples are presented in Table 6. Archimedes method investigations confirmed that samples printed using the PBF-LB/M technique exhibited lower porosity and consequently higher density. These values amounted to 4.51% and 7.397 g/cm^3^, respectively. The density of MEX samples was 7.356 g/cm^3^. Compared to the PBF-LB/M sample, it was lower by just under 1%, while the porosity was higher by 0.52%, reaching 5.03%.

This research indicates that, although 17-4 PH steel produced using the PBF-LB/M technique exhibits slightly lower hardness, the difference is not significant. Conventionally manufactured 17-4 PH steel demonstrates lower porosity compared to other materials intended for printing, such as 316L steel. In a study conducted by Gong et al. [27], samples produced using the MEX technique showed porosity that was greater by 1.5% compared to samples manufactured using the PBF-LB/M technique.

### 3.4. Microhardness Analysis

Microhardness tests for MEX and PBF-LB/M samples were also conducted for two planes. The applied load during the HV0.1 testing was approximately 1.0 N. The results are presented in Table 7.

Samples printed using the MEX technique exhibited higher microhardness. The results for this method were more consistent than those for the PBF-LB/M technique. Microhardness values for the XY plane were 303 HV0.1 for MEX samples and 285 HV0.1 for PBF-LB/M samples (a decrease of 6% compared to MEX samples). However, the difference was more pronounced for the YZ plane, amounting to 19%. The value for MEX was 328 HV0.1, while for PBF-LB/M, it was 267. Despite higher porosity, 17-4 PH steel printed using the MEX method achieves higher hardness. Such a phenomenon results from the fact that 17-4 PH steel is a material for solution annealing and aging. Samples obtained by means of the PBF-LB/M technique were analyzed in as-build conditions, when the samples obtained via MEX technique were debinded and sintered (which is a part of the production process of fully metallic parts). The lower hardness of SLM-made samples could be caused by the lack of additional heat treatment (solution annealing and aging), which explains the differences in hardness in favor of MEX. Furthermore, the layered structure of the SLMed parts may also affect hardness, which was taken into account in previous research [33]. Comparing the results to other previous research and available literature [30,34,35,36,37], 17-4 PH steel achieves higher hardness than 316L. What is more, there are significant differences between the hardness of PBF-LB/M and MEX samples made of 17-4PH, while in the case of 316L steel, the values were at almost the same level.

The differences in structural properties that occurred in 17-4PH steel obtained by means of PBF-LB/M and MEX techniques are mostly related to the types of annealing that the samples were subjected to. Such postprocessing affects the microstructure, which leads to further modifications in the wide performance properties of the obtained parts.

### 3.5. Roughness

The results of surface roughness measurements for the samples are presented in Table 8. It includes not only the obtained parameter values, but also images of the examined profiles. The non-blue points (x) denote the total length of the measured profiles, while the red line indicates the length used to calculate the surface roughness. The MEX samples exhibited lower Ra roughness, measuring 2.71 μm and 4.24 μm for the upper and lateral planes, respectively. In contrast, the roughness was higher for the PBF-LB/M samples for both planes. For the upper plane, the Ra roughness was three times higher, reaching 8.27 μm, while for the lateral plane, it was 5.14 μm (an increase of 17%). This research indicates that 17-4 PH steel printed using the MEX technique achieves lower roughness without additional processing. According to common procedures for parts manufactured using metal AM, these samples may require additional surface treatment, such as sandblasting, to achieve a uniform surface finish, regardless of the printing direction [38,39]. Comparing the results with those of other authors [26], 17-4 PH steel printed using the MEX technique exhibits lower roughness than 316L steel. The research results show that the surface roughness Ra of samples made from MEX 316L metal was 7.5 µm, which was significantly higher than the surface roughness of PBF-LB/M samples, which reached 5.8 µm.

## 4. Conclusions

The conducted analysis revealed microstructural and mechanical differences between two 3D printing methods used for 17-4 PH steel: MEX and PBF-LB/M. The research findings indicate that while PBF-LB/M specimens exhibit better microstructural properties, they have inferior mechanical characteristics, including hardness and surface roughness, compared to MEX counterparts. The key conclusions from the investigation are as follows:

1. Samples produced using the PBF-LB/M method had significantly lower porosity than MEX samples in both the XY and YZ planes.
2. The density of PBF-LB/M samples was much higher than that of MEX samples, indicating better structural homogeneity and fewer defects.
3. MEX specimens showed higher microhardness values than PBF-LB/M specimens.
4. MEX samples had lower surface roughness compared to PBF-LB/M samples, suggesting differences in surface quality between the two manufacturing methods.

Despite some imperfections, such as porosity, the PBF-LB/M technique demonstrated superior density and microstructural accuracy compared to MEX. However, with the increasing accessibility and ongoing improvements in MEX technology, including cost reductions and advancements in the technique, further research into its applications and defect mitigation strategies is needed to enhance its competitiveness with methods like PBF-LB/M.

## Figures and Tables

**Table 1:** Printing parameters for MEX samples.

Filament Diameter [mm]	Layer Thickness [mm]	Nozzle Diameter [mm]	Nozzle Temperature [°C]	Print Bed Temperature [°C]	Feed Rate [mm/s]	Infill [%]	Number of Contours	Infill Pattern	Cooling Speed [mm/s]	Printing Time for 3 Samples [h]
1.75	0.1	0.2	250	100	35	100	5	concentric	0	3

**Table 2:** 17-4 PH steel chemical compositions.

Elements											
Fe	Cr	Ni	Cu	Mn	Si	Nb + Ta	C	N	O	P	S
Weight (%)											
Bal.	15.00–17.50	3.00–5.00	3.00–5.00	1.00	0.07	0.15–0.45	0.07	0.10	0.04	0.040	0.015

**Table 3:** Printing parameters for PBF-LB/M samples.

Layer Thickness [mm]	Hatch Distance [mm]	Scanning Speed [mm/s]	Power [W]	Energy Density [J/mm^3^]	Printing Time for 3 Samples [h]
0.03	0.12	800	200	69.4	4.5

**Table 4:** Composition of acetic glycerol digestion solution.

Reagent	HCl	HNO_3_	CH_3_COOH	glycerol
**Quantity [mL]**	6	4	4	0.2

**Table 5:** Number of grain pores and pore volume results of the samples.

Sample	Number of Grain Pores	Pore Volume (mm^2^)	Pore Volume (%)	The Average Size of an Individual Grain (mm^2^)
MEX (XY)	2388 ± 733	5726 ± 1835	7.8 ± 2.5	4.5 ± 3.6
PBF-LB/M (XY)	1709 ± 582	2073 ± 1998	2.8 ± 2.7	1.6 ± 1.1
MEX (YZ)	2211 ± 868	4733 ± 1257	6.4 ± 1.7	2.4 ± 1.1
PBF-LB/M (YZ)	890 ± 364	1988 ± 294	2.7 ± 0.4	2.7 ± 0.8

**Table 6:** Density and porosity results of the samples.

Type of Test Sample	Density (g/cm^3^)	Porosity (%)
Conventional	7.746 ± 0.180	~0
PBF-LB/M	7.397 ± 0.139	4.51
MEX	7.356 ± 0.059	5.03

**Table 7:** Microhardness measurements.

Type of Test Sample	HV0.1 (XY)	HV0.1 (YZ)
MEX	303 ± 2	328 ± 8
PBF-LB/M	285 ± 19	267 ± 11

**Table 8:** Surface roughness measurements.

Sample Type	MEX—Upper Plane	MEX—Lateral Plane	PBF-LB/M—Upper Plane	PBF-LB/M—Lateral Plane
Surface image with the indicated profile line	* 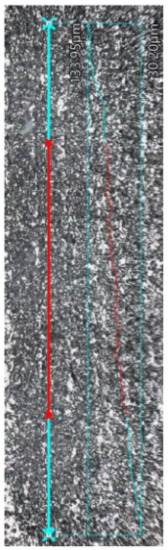 *	* 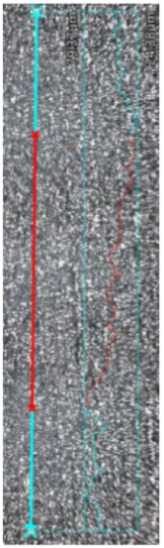 *	* 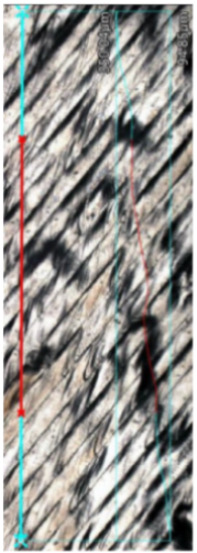 *	* 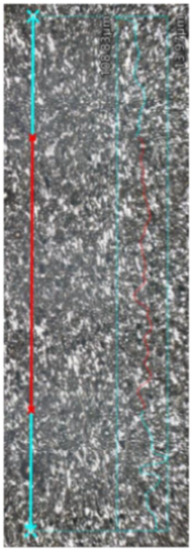 *
Measured profile length [μm]	4673.66	4751.61	4819.71	4718.80
Measured profile height [μm]	103.75	54.98	255.19	94.86
R_z_ [μm]	15.20	24.02	33.05	32.10
R_a_ [μm]	2.71	4.24	8.27	5.14

## Data Availability

The data are available upon request.
